# Supplementary material for: Collective dipole effects in ionic transport under electric fields
Source: Nat Commun. 2020 Jul 3;11:3330. doi: 10.1038/s41467-020-17173-w (PMC7335081; doi:10.1038/s41467-020-17173-w)
Supplement: Supplementary file 1 — Supplementary Information [file 41467_2020_17173_MOESM1_ESM.pdf]

Supplementary Information
Collective dipole effects in Ionic Transport under electric fields
N. Salles et al.

(a) (b)

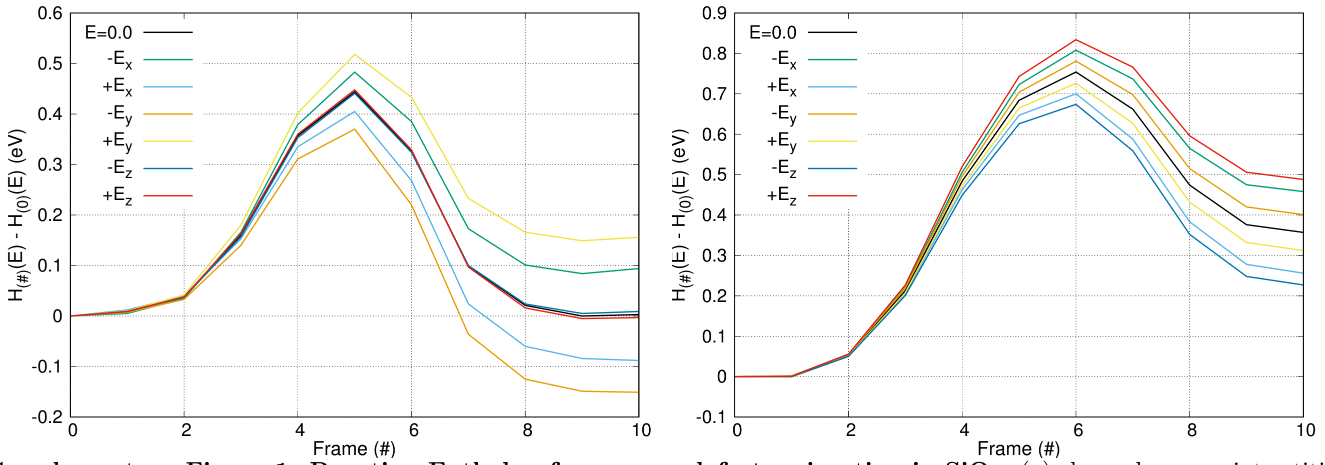

Supplementary Figure 1. Reaction Enthalpy for oxygen defects migration in SiO_2. (a) charged oxygen interstitial (I_O^{2-}) and (b) charged oxygen vacancy (V_O^{2+}). Each NEB is composed by nine intermediate frames between the initial and the final configuration. The reaction path do not include a potential further relaxation due to the field. The magnitude of the electric field is 0.001 Ry (3.6MV.cm^{-1}), twice the typical field value in a OxRRAM with thickness of tens nanometers.

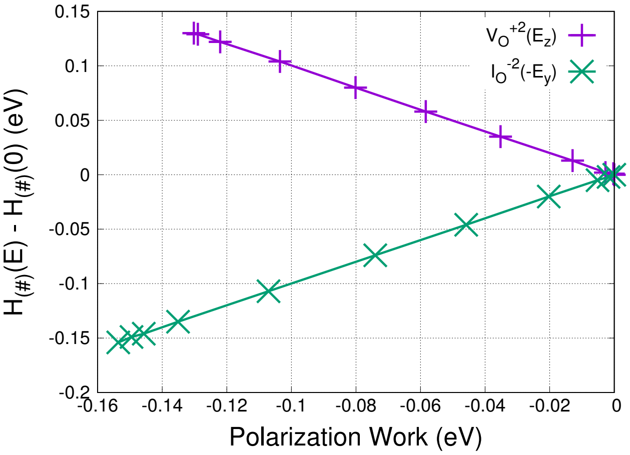

Supplementary Figure 2. Enthalpy difference along the migration path, with and without the field. Two cases, a charged -2 oxygen interstitial (I_O^{2-}) with electric field applied on $\vec{z}$ direction and a charged +2 oxygen vacancy (V_O^{2+}) with electric field applied on $-\vec{y}$ direction as a function of the polarization work.

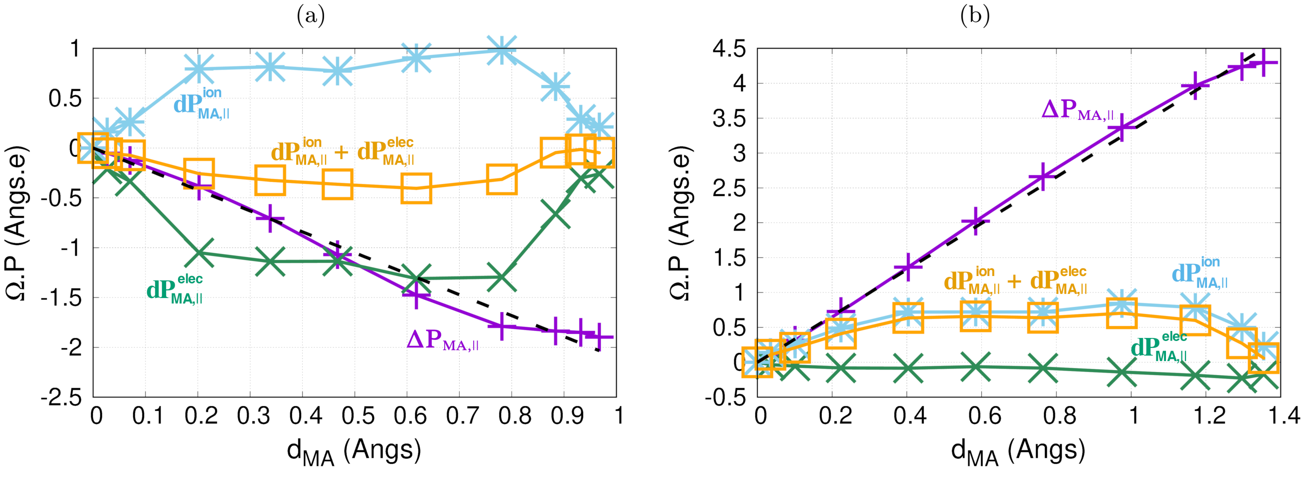

Supplementary Figure 3. Detail of electronic and ionic contribution to the ΔP^I_{MA}. The MAis an oxygen (a) I_O^{2-} and a silicon in (b) V_O^{2+} case in SiO_2 and in purple plus, their electronic $\delta\vec{P}^I_{elec,def}$ (green cross) and ionic $\delta\vec{P}^I_{ion,def}$ part (cyan star) of vector $\delta\vec{P}^I_{def}$ projected on the $\vec{u}_{\parallel}$ and their sum which correspond to the $|\delta\vec{P}^I_{def}|$ (yellow square) for each picture I of the NEB. A linear regression passing at origin is provided on the defect polarization evolution and we obtain a slope of -2.1 for I_O^{2-} and 3.3 for V_O^{2+}.

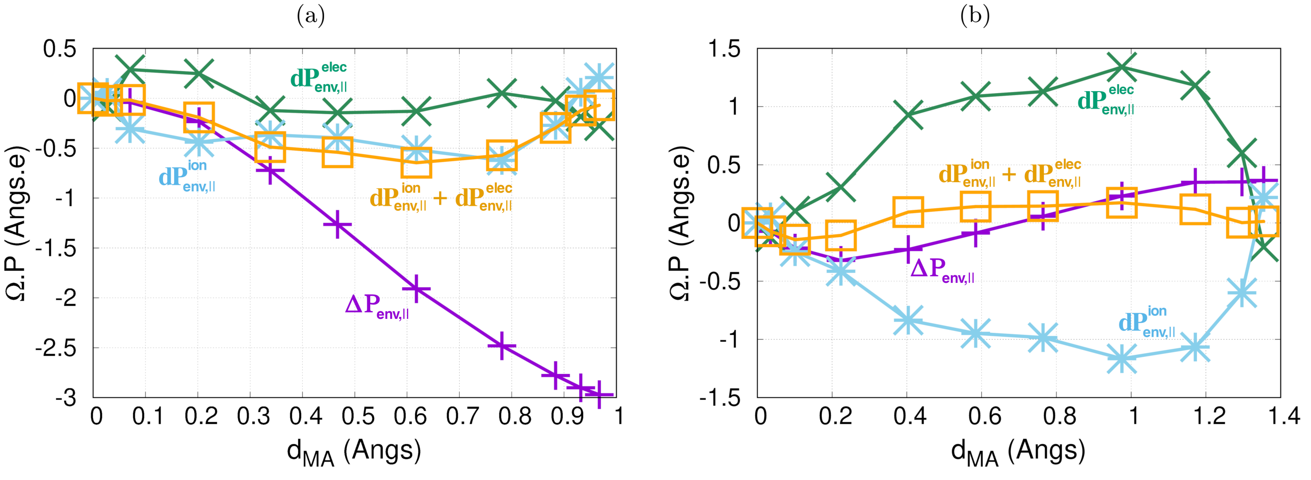

Supplementary Figure 4. Detail of electronic and ionic contribution to the environment polarization $\Delta P^I_{\mathrm{env}}$. The $\Delta P^I_{\mathrm{env}}$ is in purple plus for the (a) I_O^{2-} and (b) V_O^{2+} case, their electronic $\delta\vec{P}^I_{elec,\mathrm{env}}$ (green cross) and ionic $\delta\vec{P}^I_{ion,\mathrm{env}}$ (cyan star) part of vector $\delta\vec{P}_{\mathrm{env}}$ projected on the $\vec{u}_{\parallel}$ and their sum which correspond to the $|\delta\vec{P}^I_{\mathrm{env}}|$ (yellow square) for each picture I of the NEB

SUPPLEMENTARY NOTE 1

Electric field effect on reaction path Enthalpies for I_O^{-2}@SiO_2 and V_O^{+2}@SiO_2

As a function of the electric field direction, Supplementary Figure 1, shows that the polarization work depends on the angle between the polarization and the field, i.e. the three possible directions for the electric field (x,y,z) give different work contributions as the work if the scalar product between the polarization and the field.

For an applied electric field along y and z of 3.6 MV cm^{-1}, the energy change at the saddle point is of about ±0.08 eV, for I_O^{-2} and V_O^{+2}, respectively.

The variation of the reaction Enthalpy (the Enthalpy difference at the saddle point, with and without the electric field) is, as expected, simply, the polarization work from the initial configuration to the saddle point configuration. Indeed, in Supplementary Figure 2, a linear dependence with a slope equal to unity is obtained for the relation between the enthalpy variation and the polarization work.

SUPPLEMENTARY NOTE 2

Moving Atom and Environment contributions

To disentangle the contributions coming from a specific Moving Atom (MA) from its moving environment (env), and to numerically integrate the polarization work integral, we proceed as follows. We first discretized the reaction path into a series to frames (frame 1 is the initial configuration). The total macroscopic polarization could be written as:

$$\begin{aligned} W_{0I} &= \Omega\{\sum_{J=1}^{I} d\vec{P}^{J}\} \cdot \vec{E} \\ &= \Omega\{\sum_{J=1}^{I} d\vec{P}^{J}_{MA} + d\vec{P}^{J}_{env}\} \cdot \vec{E} \\ &= \Omega|\sum_{J=1}^{I} d\vec{P}^{J}_{MA} + d\vec{P}^{J}_{env}| \cdot |\vec{E}| \cdot cos < \Delta\vec{P}^{I}\vec{E} > . \end{aligned} \tag{1}$$

The sum over the infinitesimal polarization $d\vec{P}^J$ runs from the initial configuration 1 to a given configuration (point) J along the reaction path. The Born Effective Charge (BEC) of the an atom i, $Z^*_{i,\alpha\beta}$ is defined as the variation of the total polarization, δP_α, with respect to the variation of its positions, $\delta r_{i,\beta}$:

$$Z^*_{i,\alpha\beta} = \frac{\Omega}{|e|}\frac{\delta P_\alpha}{\delta r_{i,\beta}}, \tag{2}$$

with Ω being the volume of the simulation box. The BEC is, in general, a non-symmetric matrix.

Equation 2, can be exploited to define a macroscopic and atom-related polarization variation along the reaction path as a linear interpolation between two frames, I and $I+1$, $d\vec{P}^{I+1}_{MA}$:

$$d\vec{P}^{I+1}_{MA} = \bar{\bar{Z}}^{*(I)}_{MA} \cdot d\vec{r}^{I+1}_{MA}, \tag{3}$$

where , $d\vec{r}^{I+1}_{MA} = \vec{r}^{I+1}_{MA} - \vec{r}^{I}_{MA}$ ($I = 0$ would correspond to the starting configuration).

Along the reaction path, the total atom-related macroscopic polarization, becomes:

$$\Delta\vec{P}^{I}_{MA} = \sum_{J=1}^{I} d\vec{P}^{J}_{MA}. \tag{4}$$

From the MA polarization change, $d\vec{P}^J_{MA}$, for each frame J, the environment contribution to the polarization can be simply extracted as:

$$d\vec{P}^{J}_{env} = d\vec{P}^{J} - d\vec{P}^{J}_{MA}, \tag{5}$$

and similarly for the total environment-related polarization, $\Delta\vec{P}^I_{env}$.

To analyze the different contributions of the total macroscopic polarization we define, for each frame, a basis $\mathcal{U}^I = \left\{u^I_\parallel, u^I_\perp, u^I_\times\right\}$. $u^I_\parallel$ is defined as:

$$\vec{u}^{I}_{\parallel} = \frac{\vec{d}^{I}_{MA}}{|\vec{d}^{I}_{MA}|}, \tag{6}$$

which is parallel to $\vec{d}^I_{MA}$ ($\vec{d}^I_{MA} = \sum_{J=1}^{I} d\vec{r}^J$). In the same way we define $\left\{u^J_\perp\right\}$ as the direction parallel to $\Delta\vec{P}^I_\perp$ obtained from $\Delta\vec{P}^I_\parallel$ as:

$$d\vec{P}^{J}_{\perp} = d\vec{P}^{J} - (d\vec{P}^{J} \cdot \vec{u}^{I}_{\parallel}) \cdot \vec{u}^{I}_{\parallel}. \tag{7}$$

The two first vectors define a plane which contains the movement of MA and the polarization change of frame J. The third one is taken as the perpendicular of this plane through the cross product between the two first basis vectors:

$$\vec{u}^{I}_{\times} = \vec{u}^{I}_{\parallel} \wedge \vec{u}^{I}_{\perp} \tag{8}$$

Therefore, the magnitude of the projected quantities along $\vec{u}^I_\times$ accounts for their deviations with respect to the plane defined by the total polarization change and the MA movement.

Projecting on the basis $\mathcal{U}$, the polarization part of Equation 1 becomes:

$$\begin{aligned} &\|\sum_{J=1}^{I}\{d\vec{P}^{J}_{MA} + d\vec{P}^{J}_{env}\}\| = \\ &\qquad \|\sum_{J=1}^{I}\{(dP^{J}_{MA,\parallel} + dP^{J}_{env,\parallel}) \cdot \vec{u}^{I}_{\parallel} + \\ &\qquad (dP^{J}_{MA,\perp} + dP^{J}_{env,\perp}) \cdot \vec{u}^{I}_{\perp} + \\ &\qquad (dP^{J}_{MA,\times} + dP^{J}_{env,\times}) \cdot \vec{u}^{I}_{\times}\}\|, \end{aligned} \tag{9}$$

If the projection along the MA displacement vector carries the major part of the total macroscopic polarization, Equation 9 could be simplified as:

$$\Delta\vec{P}^I \sim \sum_{J=1}^{I}(dP^J_{MA,\parallel} + dP^J_{env,\parallel}), \tag{10}$$

been $dP^J_{MA,\parallel}$ and $dP^J_{env,\parallel}$ either positive or negative depending on the sign of their respective projection along $u_\parallel$.

SUPPLEMENTARY NOTE 3

Ionic and electronic contributions

Each polarization component can be further decomposed into ionic and electronic parts. If, from the BEC tensor, the nuclear charge, Z^{ion}, (or pseudo-atom charge in the context of pseudo-potential first-principle approaches) of the MA is removed, the remaining charge can be associated to the effective number of electrons surrounding the MA:

$$Z^*_{MA,\alpha\beta} - Z^{ion}_{MA}\delta_{\alpha\beta} = \frac{\Omega}{|e|}\frac{\delta P_\alpha}{\delta r_{MA,\beta}} - Z^{ion}_{MA}\delta_{\alpha\beta} = Z^{*\,elec}_{MA,\alpha\beta} \tag{11}$$

where $\delta_{\alpha\beta}$ is the Kroneker's delta, Z^{ion}_i is ionic charge of ion i, which is positively defined, and $Z^*_{MA,\alpha\beta}$ is the BEC associated to the MA. The Greek letters are used for the Cartesian axes indices. Therefore, the electronic contribution to the total polarization variation, $dP_{elec,\alpha}$ could be calculated from:

$$dP^{elec}_{MA,\alpha} = \sum_\beta \frac{|e|}{\Omega} Z^{*\,elec}_{MA,\alpha\beta}.dr_{MA,\beta}. \tag{12}$$

Consequently, $d\vec{P}^{elec}_{env}$ can be find from:

$$d\vec{P}^{elec}_{env} = d\vec{P}^{elec} - d\vec{P}^{elec}_{MA}, \tag{13}$$

and similarly for the ionic contribution.

Two different behaviors are observed for both the I^{2-}_O and the V^{2+}_O, see Supplementary Figures 3 (a) and (b), and Figures **??** (a) and (b). For the I^{2-}_O case, ionic and electronic parts contribute to the MA polarization with opposite signs, being the electronic contribution slightly higher. On the contrary, for the V^{2+}_O case, only the ionic part is relevant and accounts for almost the overall total MA polarization. So, the nature of the moving charge is in the first case electronic while in the second ionic, as expected from the physico-chemistry of both mechanisms.

On the environment side, Supplementary Figure 4 (a) and (b), the behavior is opposite: mainly ionic in the I^{2-}_O case while slightly more electronic in the V^{2+}_O case. Such alternating behaviors between the MA and environment macroscopic polarization are indications of compensations between the species.
